# Pharmacist and patient experiences of primary care during the COVID-19 pandemic: An interview study

**DOI:** 10.1016/j.rcsop.2022.100193

**Published:** 2022-10-13

**Authors:** Laura L. Gleeson, Aoife Ludlow, Barbara Clyne, Ben Ryan, Rob Argent, James Barlow, Lisa Mellon, Aoife De Brún, Muriel Pate, Ciara Kirke, Frank Moriarty, Michelle Flood

**Affiliations:** a School of Pharmacy and Biomolecular Sciences, RCSI University of Medicine and Health Sciences, 123 St Stephen's Green, Dublin 2, Ireland; b Department of General Practice, RCSI University of Medicine and Health Sciences, 123 St Stephen's Green, Dublin 2, Ireland; c Department of Pharmaceutical and Medicinal Chemistry, RCSI University of Medicine and Health Sciences, 123 St Stephen's Green, Dublin 2, Ireland; d Department of Health Psychology, School of Population Health, RCSI University of Medicine and Health Sciences, 123 St Stephen's Green, Dublin 2, Ireland; e UCD Centre for Interdisciplinary Research, Education and Innovation in Health Systems (UCD IRIS), School of Nursing, Midwifery & Health Systems, University College Dublin, Dublin 4, Ireland; f National Quality and Patient Safety Directorate, Health Service Executive, Dr Steevens' Hospital, Dublin 8, Ireland

**Keywords:** COVID-19, Primary care, Medication safety, Pharmacy, Patient experiences

## Abstract

**Introduction:**

A number of significant changes designed to reduce the spread of COVID-19 were introduced in primary care during the COVID-19 pandemic. In Ireland, these included fundamental legislative and practice changes such as permitting electronic transfer of prescriptions, extending duration of prescription validity, and encouraging virtual consultations. Although such interventions served an important role in preventing the spread of infection, their impact on practice and patient care is not yet clear. The aim of this study was to investigate patient and healthcare professional (pharmacist and general practitioner) experiences to understand the impact of COVID-19 on primary care and medication safety during the first two years of the COVID-19 pandemic in Ireland.

**Methods:**

A qualitative study using semi-structured interviews was undertaken between October 2021 and January 2022. Participants included twelve patients, ten community pharmacists, and one general practitioner. Interviews were transcribed verbatim and analysed using thematic analysis. Only patient and pharmacist interviews were included.

**Findings:**

Themes from the interviews included: 1) Access to care, 2) Technological changes, 3) Experiences of care, and 4) Patient safety. Particular challenges identified included the difficulty faced by patients when accessing care, impacts on experiences of patient care, and extensive changes to pharmacy practice during the pandemic.

**Conclusions:**

This study found that COVID-19 countermeasures considerably impacted patient and pharmacist experiences of primary care in terms of care and medication safety. While many changes were welcomed, others such as virtual consultations were received more cautiously likely due to the rapid and unplanned nature of their introduction. Further research is needed to identify how to optimise these changes to improve pharmacist and patient experience, and to understand the impact on patient safety.

## 1 Introduction

The COVID-19 pandemic had a profound impact on the delivery of healthcare around the world.^1^ Many of these changes occurred in primary care (defined in Ireland as any health or social care service provided in the community).^2^ These changes aimed to minimise face-to-face interactions and prevent the spread of COVID-19 in healthcare facilities such as general practices and community pharmacies. In many jurisdictions, primary care health professionals engaged new technology-based interventions to support delivery of care remotely as a core part of their response. Studies from Ireland, Spain and the United Kingdom reported an increase in the use of virtual consultations by general practitioners (GPs) during the pandemic.^3^, ^4^, 5. The role of the pharmacist was also extended through legislative and practice changes in many countries to relieve pressure on health systems, build capacity, and support continuity of care for patients' routine health needs. Examples of such changes around the world included introduction of electronic prescribing, authorisation to extend or renew prescriptions beyond existing periods defined in law, permitting home delivery of medicines, permitting pharmacists and pharmacy technicians to administer vaccines, and introducing virtual consultations by pharmacists.^6^

In Ireland, a number of significant changes were implemented at the beginning of the COVID-19 pandemic in 2020 to ensure patients' continued access to their ongoing treatment and to ease the additional burdens on GPs and pharmacists. Temporary amendments were made to legislation to allow for the first time the electronic transfer of prescriptions from GPs to pharmacies using Healthmail (a secure clinical email service which had previously been used solely for the transfer of patient information and communication about patient care).^7^^,^^8^ Previous to this, electronic transfer of prescriptions had not been permitted. Other amendments extended prescription validity from six to nine months and enabled pharmacists to use professional judgment to make additional supplies of prescription-only medicines according to specific requirements based on the class of medicine.^7^ From a practice perspective, GPs were encouraged to conduct patient consultations virtually where possible, and pharmacists were encouraged to organise medicines home delivery services, a service ot routinely provided by pharmacies in Ireland previously.^9^^,^^10^ While these changes served an important and timely role in helping prevent spread of infection and supporting continuity of patient care, by their nature they reduced opportunities for traditional face-to-face interaction and informal communication between patients and healthcare professionals about safe medicines use.^11^

The ‘knock-on’ impacts of these changes on patients, healthcare professionals, and public health more broadly are not yet fully understood.^12^ An initial survey study conducted with GPs and pharmacist participants in Ireland indicated that significant workflow changes occurred, with three quarters of respondents introducing Healthmail to their practice and half reporting introducing telephone consultations since the start of the pandemic.^13^ Respondents highlighted pressure, managing patient expectations, and patient monitoring as key challenges encountered. This study aimed to build on that research to explore healthcare professional and patient experiences of primary care and medication safety in more depth and gain deeper understanding of the impact of COVID-19 on primary care.

## 2 Methods

### 2.1 Study design

A descriptive qualitative methodology using semi structured interviews was considered the most appropriate study design to achieve the study aims, as it would allow the team to explore experiences of patients and healthcare professionals.^14^ Ethical approval for the study was granted by the Research Ethics Committee (REC) of the RCSI University of Medicine and Health Sciences (REC No. 202105021). The COnsolidated criteria for REporting Qualitative research (COREQ) checklist was used to guide study reporting.^15^

### 2.2 Study participants and recruitment

Study participants were recruited using a mixture of convenience and snowball sampling. Information about the study and contact details for the research team were shared on two social media platforms (Twitter and LinkedIn) from August to November 2021. Potential participants who contacted the research team were emailed a participant information leaflet and consent form that gave an overview of the study, explained that participation was voluntary, and outlined that the interviews were expected to take approximately 30 min.

Eligibility criteria for patients were being over 18 years of age and having engaged with primary care (GP or community pharmacy) to any extent since March 2020. Eligibility criteria for healthcare professionals were being a registered pharmacist/physician and being in active practice as a community pharmacist/GP in Ireland since January 2020 (to ensure participants had experience of primary care practice prior to the introduction of the COVID-19 public health measures in March 2020). Participants confirmed their eligibility and provided consent before interviews were scheduled. All eligible participants who contacted the research team took part in an interview. Participants were provided with a €25 gift voucher in recognition of their time.

### 2.3 Data collection

Semi-structured interviews were conducted over telephone or Microsoft Teams (MS Teams) by one of two researchers (LG and AL), at a time that was suitable for the participant. LG is a pharmacist and postdoctoral health services researcher; AL is an interaction designer. Both researchers are female and had training and experience in conducting qualitative interviews. Any participants known to one of the researchers were interviewed by a researcher to whom they were not known. All participants were informed of the study aim before interviews were conducted, and only one researcher was present for each interview.

Separate topic guides were developed for healthcare professionals and patients with input from the wider research team (Appendix A, Appendix B). Both topic guides were pilot tested; pilot interviews were not included in the final sample. To ensure anonymity, demographic information was not collected. Patients were asked about their experiences of accessing primary care and using medicines during the pandemic. Healthcare professionals were asked about their experience of practice during the COVID-19 pandemic, changes to their practice, the impact of the pandemic on medication safety, and the impact of the pandemic on their interprofessional working relationships. Participants were invited to provide examples of medication safety issues they had experienced. In order to allow participants to share experiences they felt were most relevant, no specific definition of medication safety was provided or used in the development of the interview questions. At the end of each interview, participants were asked if they had any further comments regarding the impact of COVID-19 on primary care, and how primary care could be improved going forward.

### 2.4 Data analysis

Files recorded using MS Teams were converted to MP3 files as soon as possible after the interviews and the MS Teams recording was deleted to preserve participant anonymity. Audio recordings of interviews were transcribed verbatim and transcripts were de-identified at this time. Patient and healthcare professional (pharmacist) interviews were analysed separately in order to ensure their experiences were fully considered, using thematic analysis as described by Braun and Clarke.^16^ Thematic analysis consists of six stages: 1) familiarisation with the data, 2) generation of initial codes, 3) searching for themes, 4) reviewing themes, 5) naming and defining themes, and 6) writing the report.^16^ Data familiarisation began while interviews were being transcribed and by reading and re-reading transcripts. QSR International's NVIVO Version 12 was used to manage the qualitative data. All interviews were coded by LG and a sample of the transcripts was coded by AL. Themes were defined and named collaboratively by both coders and MF. Patient and pharmacist themes were then compared and combined to develop the final set. The researchers conducting the analysis sought to address reflexivity by discussing their professional experience, potential biases, and preconceptions about the research area throughout the data analysis process. Both had joined the funded project after it had been designed.

### 2.5 Findings

Interviews were conducted with twelve patients and ten community pharmacists. Only one GP interview was conducted as recruitment proved difficult; this extremely low response rate meant that the interview was ultimately excluded from analysis to preserve participant anonymity. Patient interviews ranged from 10 to 30 min, with an average interview duration of 20 min. Pharmacist interviews ranged from 21 to 46 min, with an average in duration of 34 min. Four themes were developed from the patient and pharmacist interviews: 1) *Access to primary care*, 2) *Technological changes* 3) *Experiences of care* and 4) *Patient safety*. A summary of the themes with examples is provided in Table 1.

**Table 1:** Themes and summary of examples provided by participants.

Theme	Examples
Access to primary care	• Pharmacy remained available for face-to-face care while other services restricted access (Patients and Pharmacists) • Majority of GP care provided via telephone (Patients) • Nature and speed of changes resulted in additional stress (Pharmacists)
Technological changes	• Introduction of electronic transfer of prescriptions via Healthmail improved efficiency for patients and communication (Patients and Pharmacists) • Healthmail generated additional administrative burden (Pharmacists)
Experiences of care	• Telephone consultations with GPs could feel rushed or impersonal (Patients) • Pharmacy services remained similar bar capacity restrictions and physical barriers (Patients and Pharmacists) • Home medicines delivery services were introduced by pharmacies (Pharmacists)
Patient Safety	• Concern about limited access to GP review/monitoring (Pharmacists) • Workflow changes had mixed impact through initially increasing stress but subsequently meaning prescriptions were ordered ahead (Pharmacists) • Patients did not identify any safety concerns themselves

### 2.6 Access to primary care

Patient and pharmacist participants explained that the ways in which primary care was accessed changed abruptly, leading to new practices and changes to the relationship between pharmacists and patients. Patient and pharmacist participants reported that due to COVID-19 restrictions many GPs could only offer telephone consultations or were sometimes sending prescriptions without speaking to patients at certain phases of the pandemic out of necessity. Combined with the fact that many patients, particularly those at higher risk of severe COVID-19, were afraid to enter pharmacies or GP surgeries due to risk of infection, this meant that, according to the interview participants, some patients had little to no contact with healthcare professionals or monitoring throughout the pandemic.

Both patients and pharmacists noted the pharmacy as a key point for accessing face-to-face primary care services. Patients had mixed experiences accessing primacy care during the pandemic. Regarding GP care, a number of patients reported that they found it very difficult to get an appointment, and described having to explain their symptoms to a secretary to get an in-person appointment: “*They were kind of reluctant to see you, and I did find having to, in my own GP's, was having to ring up and explain to the secretary what was wrong with you”* (Patient 01). Others reported only being offered telephone consultations: “*Everything with the GP was over the phone, as such, up to yesterday when I got the flu jab for this season, I hadn't seen my GP in I'd say nearly two years. Well, probably, over 18 months anyway”* (Patient 05). In contrast, a smaller number of patients did not feel that their access to care changed during the pandemic; they saw their GP in person and had no problem getting an appointment: “*Absolutely no problems, if you needed an appointment, you rang and you got it”* (Patient 06).

These rapid changes to the accessibility of other primary care services was reported as being a particularly stressful experience for pharmacists.

*“At the beginning… it was a bit stressful… us not knowing what we could do and then patients not really knowing what way things were going to be done, um, everybody was kind of just a bit up in a heap and didn't know what was going on”* (Pharmacist 01)

Pharmacists felt very aware of being on the ‘frontline’ of the pandemic response, and the fact that pharmacies stayed open while many other primary care services had access that was more limited:

*“To be honest at the start, it was very hard going, I suppose in general pharmacies were kind of the only you know healthcare professional that like didn't have their doors closed. Like you couldn't, you know, do that. So, I suppose we were kind of taking on the brunt of GPs having their practices closed, maybe dentists having their practices closed and I suppose in that element you kind of got a lot of first hand of the stress that people were experiencing because you were the most accessible kind of healthcare professionals that they could talk to”* (Pharmacist 06)

As they were generally more accessible to the public than other primary care-based healthcare professionals during this time, pharmacists saw an increase in minor ailments consultations: *“We find that we would have a lot of people coming in for minor ailments to us rather than going to the doctor”* (Pharmacist 03). At the beginning of the pandemic, the legislation surrounding prescription repeats and emergency supplies (including for controlled drugs) was amended, which allowed pharmacists to use their professional judgment more when deciding whether to dispense a prescription due to more limited access to other routine services:

*“It probably put it into the hands of the pharmacist more as a health care provider in terms of what's reasonable… if somebody has been on methadone the same dose for maybe two or three years, I'm sure you can use your professional judgment to hand out one day without a prescription”* (Pharmacist 09)

Pharmacists generally felt that their relationships with patients improved during the pandemic, as patients saw community pharmacies as an accessible source of medical information, support, and social connection:

*“I think ‘cause pharmacies were one of the only things still open… I noticed a lot more that people would look for reassurance or maybe stay in the pharmacy longer just to maybe chat to you, whereas previously they might have just collected their medicines and left”* (Pharmacist 05)

### 2.7 Technological changes

Both pharmacist and patient participants highlighted the impact of technological changes implemented during the COVID-19 pandemic. Patients were generally positive about their experience of the implementation of electronic prescription transfer via Healthmail. Interview participants found it very practical not to have to collect a physical prescription from the GP surgery and deliver it to the pharmacy, although they were not always fully aware of the nature of the change:

*“They send it by email I think, or something, which is fantastic for both of them, I think, really, because, I had to renew a prescription there lately… so I just got on to the GP surgery straight away and they said ‘that's no problem’ and they sent over the prescription”* (Patient 04)

Pharmacist participants were also largely positive about the introduction of Healthmail.

*“Before that there was no electronic prescribing and we had the Healthmail, the secure emails and, before very few GPs would use Healthmail and they would have had to been followed up with the paper prescription before the pandemic. But then the changes in regulation meant that the GPs could send prescriptions electronically on health mail and that eliminated the need for the paper copy”* (Pharmacist 01)

Pharmacists perceived the use of Healthmail as a positive change due to improved legibility and traceability of prescriptions, and the ability to communicate with GPs via email:

*“From a work point of view obviously Healthmail is hugely beneficial, it's unbelievable… it's so efficient… ‘cause we communicate back and forth with the GPs, normally through health mail and then you have a written account of your interaction… as well as that the patient's history, it's easier to search back”* (Pharmacist 04)

However, pharmacists also found that Healthmail could increase their workload. This was due to the new associated workflow requiring a higher number of telephone calls with patients and additional paperwork:

“*We're having to print out three lots of paper or 6 months, you know if they have a medical card we're printing out six sheets of paper, so like it's all on us, the ink and the paper as well and the printer to make sure that we have one that works because there was a time there when it broke down in the middle of the pandemic and we were like all of our prescriptions are like emails that we have to print off so that was a nightmare”* (Pharmacist 03)

### 2.8 Experiences of care

Patients and pharmacists described the impact of the Covid-19 changes on their experiences of care. Patients were generally satisfied with their community pharmacy service throughout the pandemic; while some pharmacies implemented queueing systems or asked patients to order their prescriptions in advance; most patients did not notice any major changes:

Patients also had mixed perceptions of care they received during the pandemic in primary care GP and pharmacy services. When patients saw their GP in person, they tended to have positive attitudes about their experience: “*They asked me would I like a telephone consult and I said no, I'd actually prefer to come in ‘cause I wanted her to see something, and she did, in fairness, and it was fine. It was very, very comfortable. Very safe*” (Patient 01), but some found telephone consultations to be rushed: *“They're very rushed because obviously they've had 20 phone calls to make in the space of five minutes. You can't really get the information that you wanted to, I found”* (Patient 07), and impersonal: “W*hen things are being done over the phone, it's more matter of fact…it's just, you know, you have a cough, you've this, you've that, and you know, here's what I'm recommending*” (Patient 05). Some patients who pay for their GP care felt that telephone consultations were poor value for money, as they reported paying the same price for a short phone conversation as they would for an in-person consultation:

“*You can't really properly talk to them and then it like costs money for just having a 10-minute conversation which is a bit silly, ‘cause like we had to pay like 40 Euro or something for her to be like ‘I think you should just stay on the same thing you're doing now and we'll talk again in a couple months'. Useless.*” (Patient 10)

Patients generally had positive attitudes towards their experiences with community pharmacists during the pandemic: “*My pharmacist is very good. She's very thorough when she's explaining any antibiotics or things like that*” (Patient 03). Others noted only minor changes to their experience.

*“Basically there's a sign on the door saying only one person in at a time, you can see if there's someone inside so you wait outside, when you got in, there was a table inside the door with the hand sanitiser on it, and you did your business and went back out again” (Patient 06)*

Similarly, pharmacists reported that while they found the uncertainty at the beginning of the pandemic difficult, many praised the public for being patient and understanding:

*“I think actually the public were very understanding, you know, and were very good to get on board with kind of ordering in advance, there wasn't as much pressure, you know, they were kind of understanding, to give us notice, that it might take a little bit longer to put things together, that, you know, they might not be able to come into the pharmacy, that we could deliver it if possible to them. From that point of view, I actually found the public way more understanding”* (Pharmacist 04).

Other changes that impacted established experience included the introduction of delivery services. Few pharmacies would generally have operated formal delivery services, and most people would be familiar with visiting the pharmacy to collect their medicine. This was therefore a commonly encountered change: “*Once people realised that we could deliver and that people would be able to drop the medication to them… then they were fine.”* (Pharmacist 08.) Telephone consultations were then needed as the medicines were being delivered by a driver or in some cases being collected by someone else on behalf of the patient. Pharmacists needed to take additional steps to ensure patients had access to advice: *“I would often say to the person who is picking up, our number's on the bag, please give us a ring, or if it was something very specific… I've just asked the patient to phone me if they have any questions at all, if there's anything there unsure of”* (Pharmacist 09).

While many patients quickly adapted to changes in their experience, pharmacists felt that some, especially older patients, found it difficult to understand the changes that occurred:

*A lot of the customers are elderly in our pharmacy and they don't understand. They found it hard to understand how prescriptions were being emailed. A lot of them would be used to going the doctor and picking up their piece of paper and seeing themselves what's written on it. And they didn't really understand how we were getting the prescriptions. And then when they weren't coming into the pharmacy, they were so used to coming into the pharmacy and seeing us and talking to us and explaining to us in person what they wanted”* (Pharmacist 01)

### 2.9 Patient safety

Several pharmacist participants expressed concerns about how the COVID-19 countermeasures impacted patient safety, including more limited access to their GP for routine care.

*“There have been quite a few issues where people maybe have stopped attending their GP, even just to have their kind of review every six months or every three months, even just to check in… it kind of maybe means that there are issues that aren't followed up with… it makes you worry that there could be medication changes or issues that are not being kind of attended to”* (Pharmacist 05)

Pharmacists were also concerned about transitions of care and the lack of communication between hospitals, GPs and community pharmacies, which could potentially lead to medication errors: *“Communication between hospital, pharmacy and GP… we just get the prescriptions and we're expected to just do it without being told that it's an addition”* (Pharmacist 03).

The other significant patient safety challenge that emerged during the pandemic was the constant pressure that pharmacists were under due to higher workloads, which could increase the risk of medication errors. Pharmacists mentioned slowing down and performing double checks as medication safety strategies they used during the pandemic: *“I've definitely slowed myself down and I've tried to become more careful… I've become very cognizant of being extra careful because of the level of work that's been there and the pace of it”* (Pharmacist 07).

However, some pharmacists indicated that some changes enhanced patient safety. For example, many pharmacies asked patients to order their prescriptions in advance, which had a significant impact on workflow because it allowed work to be planned:

“*People kind of got much more used to like bringing in and ordering scripts, so you could plan work, which is a positive change overall to be honest with you… and obviously safer ‘cause you have more time to get stuff done*” (Pharmacist 08)

What patients understood by the questions relating specifically to medication safety differed among those interviewed. When asked what they did to ensure they took their medicines safely, most patients mentioned medicines storage:

“*I take my cholesterol one every night and we leave the packets upstairs next to the sink so, that's it sure there's only the two of us really here, you know, so there's no safety concerns*” (Patient 04) or adherence: “*I suppose, just, like, storing them at home. I just would have them somewhere where I can be reminded to take them and just, every day, the thing I find difficult is to remember to take them*” (Patient 01)

All patient participants spoke in general terms about medication safety and none identified anything specific relating to the management of their medicines during the COVID-19 pandemic.

## 3 Discussion

This study explored patient and pharmacist experiences of primary care during the COVID-19 pandemic. Our findings suggest that patients and pharmacists had mixed perspectives on how they accessed and experienced care, both patients and pharmacists identified technological changes as largely positives, and pharmacists identified concerns about patient safety but patients did not. For community patients and pharmacists, the pandemic was a period of significant change and upheaval. For pharmacists, after an initial particularly stressful period of rapid legislative and practice changes, a number of key workflow adjustments occurred, which were met with both criticism and praise. Ultimately, pharmacists felt the pandemic had a positive impact on both their role in primary care, although it was associated higher workloads and stress levels.

Both pharmacists and patients expressed concerns about access to care and patients' lack of routine engagement with primary care throughout the pandemic. Pharmacists' concern regarding access to care reflects findings from other studies.^17^ Some patients reported that they found it difficult to access their GP; when they did secure an appointment, it was often a telephone consultation, which some patients found comparatively brief and impersonal. A recent scoping review reported engagement with telehealth had both negative and positive consequences for primary care service delivery, including reduction of access to care, particularly for certain vulnerable groups such as older people.^1^ Other factors leading to reduced access to care include a combination of COVID-19 countermeasures, an overwhelming demand on primary care services, and staff shortages due to illness leading to reduced capacity.^1^ Additionally, pharmacists were concerned that patients, especially those with chronic healthcare conditions, were going for extended periods of time without being reviewed by a GP, which could result in missed diagnoses or medication errors reflecting a patient safety concern. Reduced access to primary care during the pandemic has been reported in several countries, including Belgium, Australia, and the UK.^18^, ^19^, ^20^ The difficulties encountered by patients with chronic illnesses, such as Human Immunodeficiency Virus (HIV) and diabetes, when accessing care during the pandemic have also been reported.^1^ Patient monitoring is a key aspect of chronic disease management, and poor monitoring can lead to reduced disease control and patient harm.^21^ Efforts to increase hospital capacity during the pandemic led to advances in remote patient monitoring, however the findings of this study indicate that these advances have not yet been introduced widely in primary care.^22^

An interesting finding in this study was the variability in patients' understanding of the term ‘medication safety’. When asked what strategies they used to ensure they took their medicines safely, many mentioned medicines storage or strategies to improve adherence. Patient participants also reported that they would not read the PIL of a new medicine, and trusted their doctor and pharmacist that the medicine dispensed to them was safe and correct. Medication error is a leading cause of preventable harm worldwide, so it was noted that the patients interviewed did not recognise the well-established primary care practices that were contributing towards their safe care had undergone significant change and could have been adversely impacted.^23^^,^^24^ Conversely, as would be expected, pharmacists were acutely aware of the potential impacts of the pandemic on medication safety, particularly because of increased workloads and stress levels in community pharmacy. Stress has long been recognised as a key factor in patient safety events, and the increased levels of stress and burnout experienced by frontline healthcare staff during the pandemic are well documented.^25^^,^^26^ A recent survey study by this research group found that 39% of pharmacists and 35% of GPs surveyed reported an increase in medication safety incidents during the pandemic.^13^ Findings from this study suggest that for pharmacists, workflow changes and stress may have been potential contributory factors to this increase in incidents perceived by our participants. Further research is needed on the impact of COVID-19 on medication safety incidents in primary care.

The study revealed interesting insights into the potential role of technology in primary care going forward. Patient participants in this study were generally happy about the use of Healthmail to transfer prescriptions from their perspective; although they had limited understanding of the work it generated for pharmacists, but had mainly negative attitudes towards virtual consultations. Pharmacists tended to have positive attitudes towards Healthmail; however, several issues were reported, such as an increase in workload due to paperwork and a high number of phone calls related to Healthmail prescriptions. A recent rapid review by this research group found evidence for a potential association between the electronic transfer of prescriptions and an increased rate of medication safety incidents.^27^ While virtual consultations can be an important method of primary care delivery for some patients, both pharmacists and patients interviewed in this study stated that they could not act as a replacement for face-to-face care. A qualitative study published in 2020 found that GPs had difficulty conducting patient consultations over the phone, despite a continued focus on patient-centred care.^20^ Studies on the use of virtual consultations in primary care during COVID-19 found that patients preferred telephone consultations to video consultations, patients tend to prefer in-person care to virtual consultations, and medications are more likely to be prescribed in face-to-face consultations than in virtual consultations.^28^, ^29^, ^30^ A number of rapid and significant changes in the use of technology for patient care occurred during the COVID-19 pandemic, and while many have shown good acceptability among patients and healthcare professionals, the long-term impact on patient care is not yet clear.^13^^,^^22^^,^^31^

Notwithstanding these problems identified by participants and reflected in the recent literature, it is important to note that the introduction of virtual consultations in the context of the COVID-19 pandemic occurred during a global emergency. There was extremely limited time for planning, training, or optimisation of workflow, with no clear idea how long the pandemic might persist. Therefore, the introduction not in the context of a considered change management process building on the well-established evidence base for virtual care, but a crisis response was far from ideal. When possible, the potential role of technology should be reviewed and re-evaluated. As Bashshur and colleagues note ‘*With the single exception of a physical examination, quality of care in telemedicine should be the same or no lesser than in-person care; the care process must not be short changed or compromised in any way that jeopardizes patient safety*’.^32^

### 3.1 Implications

This study described the collection and analysis of formal accounts of patient and pharmacist experiences of changes to primary care to gain a deeper understanding of the impact of COVID-19 in primary care. While this has merit in its own right, it is also important to consider what implications these findings may have for future practice. The rapid and unplanned nature of the changes reportedly resulted in a stressful experience for pharmacists, confusion for patients, and concerns about patient safety. Formal contingency planning done collaboratively with primary care professionals and patient organisations may reduce the impact of such events in future. Education and training for primary healthcare professionals may help them manage crises with less experience of stress, and they may be scope to consider its inclusion as a core competency for graduates in the future. The rapid introduction and widespread acceptance of new technologies indicates that there may be more appetite for innovation in primary care than previously anticipated from both patients and healthcare professionals. This may be leveraged further to expedite the implementation of other eHealth initiatives in primary care. Pharmacists and patients described having strong, established relationships with each other that should support implementation of new technologies and other initiatives. Unlike studies from other jurisdictions that indicated pharmacists had negative experiences leaving large proportions feeling demoralised, undervalued, and even experiencing harassment,^33^^,^^34^ pharmacists and patients in our study did not describe any such experiences. This may be due to the significant trust in the profession, with community pharmacists the most trusted profession of any kind in Ireland.^35^

### 3.2 Strengths and limitations

To the best of our knowledge, this is the first qualitative study examining patient and pharmacist experiences of primary care in Ireland during the COVID-19 pandemic. The use of semi-structured interviews provided important insights into the attitudes and experiences of study participants. However, this study has some limitations. Although the initial study aim was to conduct interviews with patients, pharmacists and GPs, GP response rate was poor, presumably due to time pressure on GPs. Only one GP interview was conducted, which was excluded from analysis to maintain anonymity should their experiences and examples render them identifiable. Selection bias may have been introduced in the recruitment process, as patients and pharmacists with negative experiences may have been more likely to take part, while pharmacists worst affected by stress and high workloads may not have had time to participate. Notwithstanding this, all participants met the inclusion criteria so will therefore have had experiences and perspectives relevant to the aims of the study.

### 3.3 Future research

This study will have important implications for health services research in Ireland as it reveals both positive and negative changes that occurred in primary care during the COVID-19 pandemic. Future research should focus on the impact of the pandemic on medication safety and patient monitoring in primary care, and develop and evaluate methods to support practice and workflow changes such as the use of Healthmail and virtual consultations. The experiences of GPs during the pandemic will be instrumental to post-pandemic health services research, and should be captured as soon as possible. While this research focused on primary care, the experiences of patients and healthcare professionals in secondary and residential care settings during the pandemic should also be investigated.

## 4 Conclusions

This semi-structured interview study investigated the experiences of patients and pharmacists in Irish primary care during the COVID-19 pandemic. Patients described limited access to face-to-face care, different perceptions of quality of care, but did not describe any differences to safe use of medicines, while pharmacists highlighted the impact of rapid response to the pandemic, adapting practice, and how patient care was impacted. Most patients reported difficulty accessing some aspects of care, particularly GP services, throughout the pandemic, and pharmacists were concerned about the impact of reduced patient-healthcare professional contact on patient care and medication safety. For pharmacists, the COVID-19 pandemic triggered many changes that affected their practice, and necessitated workflow changes within pharmacies, many of which were perceived as beneficial to continue beyond the pandemic. The experiences of patients and healthcare professionals during the pandemic can provide important insights for the future of care beyond the pandemic; future research should focus on the impact of the pandemic on medication safety, and GP experiences of Irish primary care during COVID-19.

## Funding

This work was supported by a grant from the Health Research Board under the Research Collaborative in Quality and Patient Safety (RCQPS), a collaborative initiative between the Health Research Board, the Health Service Executive, National Quality Improvement Team and the Royal College of Physicians of Ireland (Grant Number RCQPS-2020-032).

## Declaration of Competing Interest

The authors declare that they have no known competing financial interests or personal relationships that could have appeared to influence the work reported in this paper.

## Appendix A Topic guide – patient interviews

1) Thank you for taking the time to take part in this interview. The COVID-19 pandemic was a difficult time for everyone, how was it for you?
2) In this study we are interested in how people engaged with primary care, such as GPs and community pharmacists, during the pandemic. What was your experience of care during the pandemic?
  • How did you find accessing your GP during the pandemic?
  • How did you find accessing your pharmacy during the pandemic?
  • Have you used new or different way to access care (e.g., video consultations, online doctors), and can you tell me about that experience?
3) Have you helped anyone else to access care during the pandemic, and if so, can you tell me about that experience?
4) In this study, we are also interested in medication safety, and how GPs, pharmacists and patients ensure people can take their medicines safely. What do you do to ensure you are taking your medicines as best you can?
  • Did you take any extra steps to help you to manage your medicines during the pandemic, and can you tell me about these?
5) Do you have any further comments on how COVID-19 has impacted your experiences of primary care, or ways we could improve how primary care is delivered going forward?
6) Is there anything I have not asked you about that you would like to share or think we should consider?

## Appendix B Topic guide – healthcare professional interviews

1) Can you tell me about how you found working as a pharmacist/GP during the pandemic?
2) It seems like pharmacy/GP service delivery changed very rapidly at the start of the pandemic. Can you tell me about how your practice has changed through the various stages of the pandemic?
  • What has been positive/negative about the changes? What would you like to keep/remove and why?
  • Which would you say has been the most significant change and why?
  • How do you think patients have experienced these changes?
  • What type of resources did you draw on/develop (e.g., training materials, new SOPs)?
  • Has the use of digital technologies impacted on your workflow since the COVID-19 pandemic? How?
3) We are also interested specifically in patient/medication safety - what impact has COVID-19 had on this from your perspective?
  • Why do you think this has been the case?
  • Can you (confidentially) share any specific examples?
  • What areas do you feel are most challenging from a patient/medication safety perspective and why?
  • Have you introduced any new strategies in your practice to manage patient/medication safety risks since COVID-19?
4) There are many different people/professions involved in primary care. Has COVID-19 changed the way you work with others as part of your role? Why did this happen?
• How has working with colleagues in primary care changed? (Specifically, GP/pharmacy)
• Has communication changed to reflect these changes? What (if anything) is different?
• What could be improved in terms of working with other healthcare professionals to promote patient/medication safety? And specifically in primary care?
• Who is responsible for patient/medication safety in your opinion? Specifically, GP, Pharmacist, Patient/carers?
5) Do you have any further comments on how COVID-19 has impacted patient/medication safety or your workflow, or suggestions for improvement of same?
6) Is there anything I have not asked you about that you would like to share or think we should consider?
